# P-136. Epidemiological Shifts in Histoplasmosis Hospitalizations Across the United States, 2012-2021: A Retrospective Analysis

**DOI:** 10.1093/ofid/ofae631.341

**Published:** 2025-01-29

**Authors:** Mohamed Barghout, Nouf K Almaghlouth

**Affiliations:** Lifespan, Warren Alpert Medical School of Brown University, Providence, Rhode Island; Warren Alpert Medical School of Brown University, Providence, Rhode Island

## Abstract

**Background:**

Histoplasmosis represents a substantial public health concern in the United States, especially prevalent in the Ohio and Mississippi River Valleys where seroprevalence rates range from 60% to 90%. Building on data indicating rising hospitalization rates from earlier studies covering 2001 to 2012, this research aims to further investigate the trends in histoplasmosis-associated hospitalizations post-2012, focusing particularly on mortality outcomes.

**Table 1:**
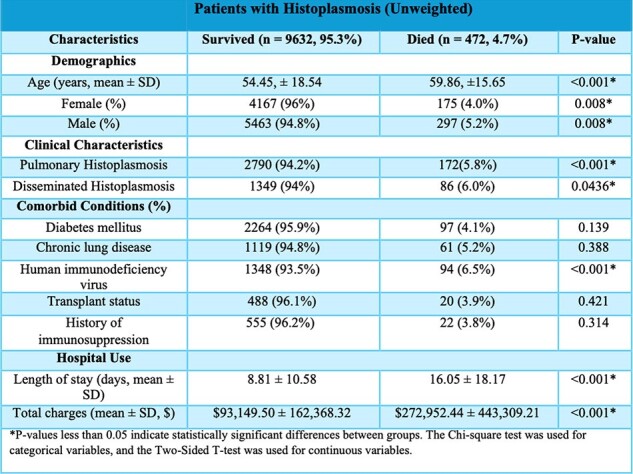
Demographic and Clinical Characteristics of Patients with Histoplasmosis (Unweighted) Categorized by Mortality

**Methods:**

We conducted a retrospective cohort study using the National Inpatient Sample (NIS) to assess trends in histoplasmosis-associated hospitalizations from 2012 to 2021. We analyzed a total of 351,321,656 hospitalizations, identifying cases of histoplasmosis either as a primary or any diagnosis. Histoplasmosis diagnoses were identified using ICD codes, and trends were analyzed using simple linear regression to calculate annual rates per 100,000 hospitalizations, with significance tested at a p-value ≤ 0.05.

**Figure 1:**
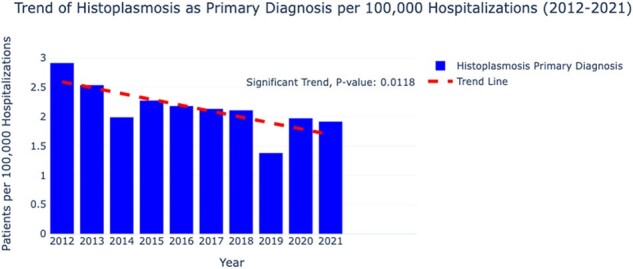
Trend of Histoplasmosis as Primary Diagnosis per 100,000 Hospitalizations (2012 - 2021)

**Results:**

There were 50,860 weighted hospitalizations with histoplasmosis listed as any diagnosis and 7,616 as the primary diagnosis over the study period. Analysis revealed a significant decline in hospitalizations, from an annual rate of 2.9 per 100,000 in 2012 to 1.9 in 2021 (p=0.0118). Patients who died were older on average compared to those who survived (59.86 years vs. 54.45 years, p< 0.001) and had significantly longer hospital stays and higher healthcare costs. The overall mortality rate was 4.7%. The most common presentations were pulmonary (5.8% died, p=0.0054) and disseminated forms (6.0% died, p=0.0436). Comorbid conditions had varied impacts on patient outcomes; particularly, patients with HIV had a higher mortality rate (6.67%, p=0.0031). Notably, an upward trend was observed in the incidence of histoplasmosis among diabetic patients (p=0.0113), while other comorbidities did not show significant trends.

**Figure 2:**
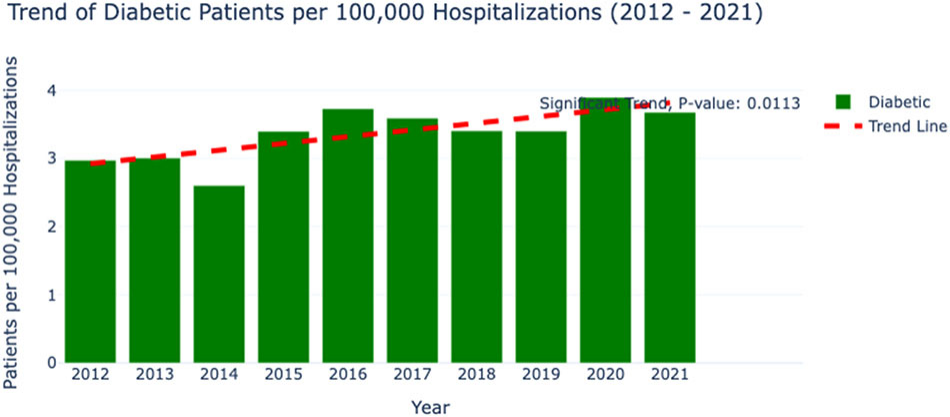
Trend of Histoplasmosis in Diabetic Patients per 100,000 Hospitalizations (2012 - 2021)

**Conclusion:**

The significant reduction in histoplasmosis-associated hospitalizations between 2012 and 2021 reflects advancements in public health initiatives and enhancements in clinical management. Nonetheless, our study highlights an increased mortality rate linked to older age, the presence of comorbidities such as HIV, prolonged hospital stays, and elevated healthcare cost.

**Disclosures:**

**All Authors**: No reported disclosures

